# Supplementary material for: Host–pathogen dynamics in longitudinal clinical specimens from patients with COVID-19
Source: Sci Rep. 2022 Apr 7;12:5856. doi: 10.1038/s41598-022-09752-2 (PMC8987511; doi:10.1038/s41598-022-09752-2)
Supplement: Supplementary file 1 — Supplementary Information. [file 41598_2022_9752_MOESM1_ESM.docx]

Supplementary Information

**Host-pathogen dynamics in longitudinal clinical specimens from patients with COVID-19**

Michelle J. Lin^1^, Victoria M. Rachleff^1,2,3^, Hong Xie^1^, Lasata Shrestha^1^, Nicole A.P. Lieberman^1^, Vikas Peddu^1^, Amin Addetia^1^, Amanda M. Casto^4^, Nathan Breit^1^, Patrick C. Mathias^1^, Meei-Li Huang^1,2^, Keith R. Jerome*^¶1,2^, Alexander L. Greninger*^¶1,2^, Pavitra Roychoudhury*^¶1,2^

^1^Department of Laboratory Medicine and Pathology, University of Washington School of Medicine, Seattle, WA, USA, 98102

^2^Vaccine and Infectious Disease Division, Fred Hutchinson Cancer Research Center, Seattle, WA, USA

^3^Program in Molecular and Cellular Biology, University of Washington School of Medicine, Seattle, WA, USA

^4^Division of Allergy and Infectious Diseases, University of Washington School of Medicine, Seattle, WA, USA

**Supplementary Table S1**. **Description of patients included in this study.**

^1^at sample collection. IP = inpatient, ER = emergency room, OP = outpatient, NH = nursing home, NP = nasopharyngeal, OP = oropharyngeal

| **Patient** | **Age** | **Sex** | **Status when tested** | **Comorbidities** | **Treatment for COVID-19** | **Sample type(s)** | **Time points** |
| --- | --- | --- | --- | --- | --- | --- | --- |
| P001 | 72 | F | NH | CLL, hypogammaglobulinemia, hypothyroidism | Hydroxychloroquine, Convalescent Plasma x 2 | Naso-  pharyngeal | 2 |
| P002 | 42 | M | Unknown | Asthma, Hypertension, Sleep Apnea | Azithromycin, ACTT-1 Trial (Remdesivir versus Placebo) | Naso-  pharyngeal | 2 |
| P003 | 49 | M | Unknown | Unknown | Unknown | Naso-  pharyngeal | 2 |
| P004 | 98 | M | IP | Lewy Body Dementia, Coronary Artery Disease, Chronic Kidney Disease, Hypertension, Heart Failure, Benign Prostatic Hypertrophy | Azithromycin | Naso-  pharyngeal, Oro-  pharyngeal | 3 |
| P005 | 90 | F | OP | Dementia, Diabetes, Hypertension |  | Naso-  pharyngeal | 2 |
| P006 | 88 | F | NH | Hypertension, Hyperlipidemia, Cardiomyopathy, Previous Stroke, Chronic Kidney Disease |  | Naso-  pharyngeal, Oro-  pharyngeal | 1 |
| P007 | 54 | M | OP / IP | Obesity, Protein S Deficiency, Hyperlipidemia, Sleep Apnea | ACTT-1 Trial (Remdesivir versus Placebo), Tocilizumab, Azithromycin | Naso-  pharyngeal | 2 |
| P008 | 80 | F | ER / IP | Hypertension, Neurocognitive Disorder with delusions | Hydroxychloroquine | Naso-  pharyngeal | 2 |
| P009 | 70 | M | ER / IP | Heart Failure, COPD, Obesity, Sleep Apnea, Chronic Kidney Disease, Diabetes, Coronary Artery Disease, | None | Naso-  pharyngeal | 2 |
| P010 | 73 | M | IP | Dementia, Benign Prostatic Hyperplasia | None | Naso-  pharyngeal | 4 |
| P011 | 80 | M | ER / IP | Diabetes, Hypertension, Hyperlipidemia, Chronic Kidney Disease | Azithromycin | Naso-  pharyngeal | 3 |
| P012 | 51 | M | ER | Chronic Hepatitis B | None | Naso-  pharyngeal | 1 |
| P013 | 44 | M | ER / IP | Seizure Disorder, Chronic Kidney Disease, Sleep Apnea, Hypertension, Hyperlipidemia | None | Naso-  pharyngeal | 2 |
| P014 | 83 | M | NH | Dementia, Hypertension, Hyperlipidemia | None | Naso-  pharyngeal | 2 |
| P015 | 78 | F | ER / IP | Mitral Stenosis, Tachy-brady Syndrome, Coronary Artery Disease, Heart Failure, Kidney Transplant, Diabetes | None | Naso-  pharyngeal | 3 |
| P016 | 98 | F | IP | Atrial Fibrillation, Sick Sinus Syndrome, Peripheral Vascular Disease, Hyperlipidemia, Hypertension, Chronic Kidney Disease | None | Naso-  pharyngeal | 2 |
| P017 | 77 | M | IP | Atrial Fibrillation, Diabetes, Hypertension, Diabetes, Stroke, Aortic Stenosis | Azithromycin | Naso-  pharyngeal | 2 |
| P018 | 69 | M | IP | Renal Failure, Multiple Myeloma, Heart Failure, Coronary Artery Disease, Chronic Obstructive Pulmonary Disease, Diabetes | Convalescent plasma, Dexamethasone, Empiric Vitamin C, Thiamine | Naso-  pharyngeal | 2 |
| P019 | 42 | F | OP | Unknown | Unknown | Naso-  pharyngeal | 2 |
| P020 | 59 | M | OP | Chronic Liver Disease/Liver Failure, Anemia, Coronary Artery Disease, Diabetes | None | Naso-  pharyngeal | 2 |

**Supplementary Figure S1**. **Viral loads for all patients included in the study with samples that returned positive (DET), negative (NDET, Ct >42), and inconclusive (INCON) result by RT-PCR.**

Red dots represent samples for which we attempted sequencing for this study. Legend above the plot matches shapes of dots to respective RT-PCR results.


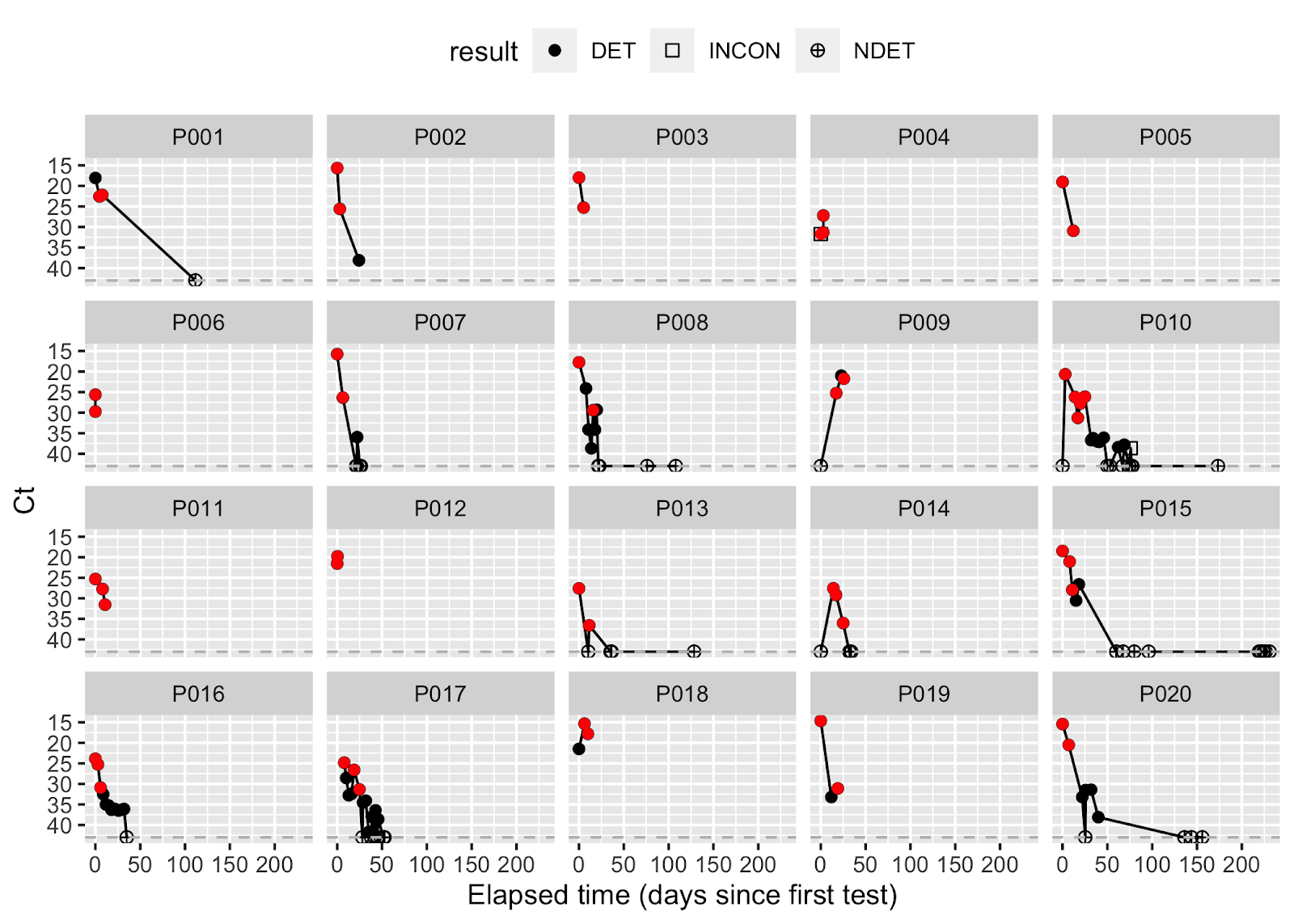


**Supplementary Figure S2**. **Sequencing quality and reproducibility.**

(A) Samples were sequenced by metagenomic, probe-capture or amplicon-based approaches. Hollow circles show samples for which a consensus genome could not be recovered. Genome completeness indicates the percentage of non-N nucleotides in consensus sequence (an N was called when there was less than 5X coverage at a site). (B) Each dot represents a coding change in a single sample relative to the Wuhan-Hu-1 (NC_045512.2) reference genome with variant allele frequency between 5-100%, at least 100x coverage at the site, and intra-host reproducibility across longitudinal samples of the same patient. Color scale represents the change in allele frequency across time points in the same patient with darker colors representing variants that had greater changes in frequency across samples. Small dark grey marks along the top margin shows positions with variant frequencies >95% (fixed mutations relative to the reference). Size of circles indicates sequencing depth at the site. Marginal histogram shows distribution of variants using bin width of 500 nucleotides.


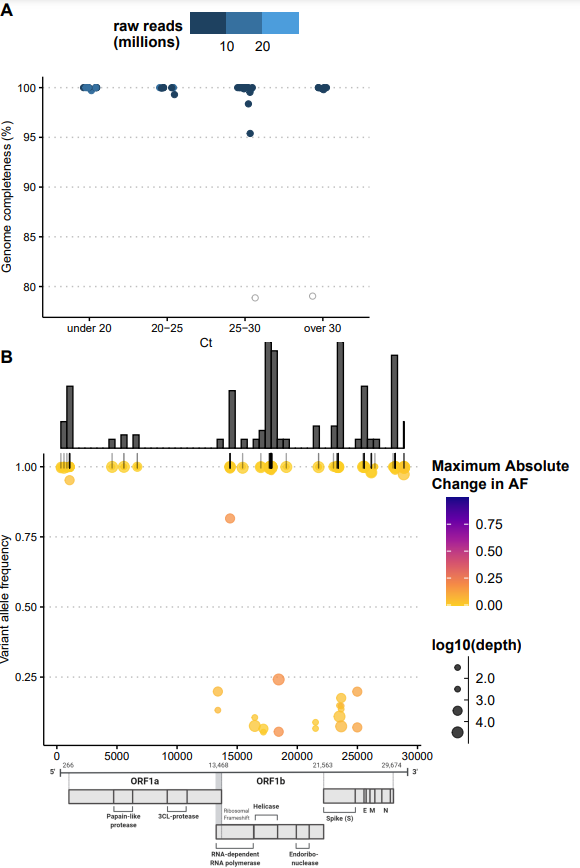


**Supplementary Table S2**. **Data availability (NCBI Bioproject PRJNA610428).**

Table of consensus sequences and their respective GISAID accession numbers and sequencing platforms. Information on whether samples were sequenced multiple times for reproducibility analysis is also included.

| **Patient** | **Sample #** | **GISAID accession** | **Sequencing platform** | **Has replicate?** |
| --- | --- | --- | --- | --- |
| P001 | 1 | EPI_ISL_792092 | MiSeq | Yes |
|  | 2 | EPI_ISL_570115 | MiSeq | No |
| P003 | 1 | EPI_ISL_416643 | NextSeq | No |
|  | 2 | EPI_ISL_418914 | MiSeq | Yes |
| P005 | 1 | EPI_ISL_427186 | NextSeq | No |
|  | 2 | NA | NextSeq | No |
| P006 | 1 | EPI_ISL_416454 | NextSeq | No |
|  | 1 | EPI_ISL_792093 | NextSeq | No |
| P007 | 1 | EPI_ISL_416448 | NextSeq | No |
|  | 2 | EPI_ISL_418942 | NovaSeq | No |
| P008 | 1 | EPI_ISL_424228 | NovaSeq | No |
|  | 2 | EPI_ISL_461429 | NextSeq | No |
| P009 | 1 | EPI_ISL_570975 | NextSeq | No |
|  | 2 | EPI_ISL_792094 | NextSeq | Yes |
| P010 | 1 | EPI_ISL_416717 | NextSeq | No |
|  | 2 | EPI_ISL_515280 | MiSeq | No |
|  | 3 | EPI_ISL_792095 | MiSeq | Yes |
|  | 4 | EPI_ISL_486097 | MiSeq | Yes |
| P011 | 1 | EPI_ISL_570059 | NextSeq | Yes |
|  | 2 | EPI_ISL_486102 | MiSeq | Yes |
|  | 3 | EPI_ISL_461405 | MiSeq | No |
| P012 | 1 | EPI_ISL_424190 | NovaSeq | No |
|  | 1 | EPI_ISL_424205 | NovaSeq | No |
| P013 | 1 | EPI_ISL_792096 | MiSeq | No |
|  | 2 | EPI_ISL_460634 | MiSeq | Yes |
| P014 | 1 | EPI_ISL_486103 | MiSeq | No |
|  | 2 | EPI_ISL_792097 | MiSeq | No |
| P015 | 1 | EPI_ISL_424262 | NextSeq | Yes |
|  | 2 | EPI_ISL_485994 | NextSeq | Yes |
|  | 3 | EPI_ISL_792098 | MiSeq | Yes |
| P016 | 1 | EPI_ISL_570071 | MiSeq | No |
|  | 2 | EPI_ISL_825015 | MiSeq | No |
|  | 3 | EPI_ISL_486110 | NextSeq | Yes |
| P017 | 1 | EPI_ISL_792099 | MiSeq | No |
|  | 2 | EPI_ISL_570078 | MiSeq | No |
| P018 | 1 | NA | NextSeq | No |
|  | 2 | NA | NextSeq | No |
| P019 | 1 | EPI_ISL_570762 | NextSeq | No |
|  | 2 | EPI_ISL_570779 | MiSeq | No |
| P020 | 1 | NA | NextSeq | Yes |
|  | 2 | EPI_ISL_570503 | NextSeq | No |

**Supplementary Table S3**. **Samples used for host gene expression analysis using RNAseq.**

| **Patient** | **Sample #** | **COVID Positivity** | **Reads Pseudo-aligned to Human Transcriptome** |
| --- | --- | --- | --- |
| P001 | 1 | repeat | 914378 |
| P002 | 1 | initial | 1458465 |
| P003 | 1 | initial | 920298 |
| P003 | 2 | repeat | 2115575 |
| P004 | 1 | initial | 11890988 |
| P007 | 2 | repeat | 2450327 |
| P008 | 1 | initial | 1113231 |
| P009 | 1 | initial | 5543628 |
| P015 | 1 | initial | 1544930 |
| P016 | 1 | initial | 1179241 |

**Supplementary Figure S3. Dynamics of metagenomically classified pathogens.** A) Summary of read classifications from CLOMP. Individual bars represent unique samples collected from each patient. Colors correspond to different taxonomic classifications. Reads mapping to SARS-CoV-2 are included in “Viral reads.” B) Longitudinal RPM values for *Moraxella catarrhalis* and *Staphylococcus aureus* detected by metagenomic analysis.

**
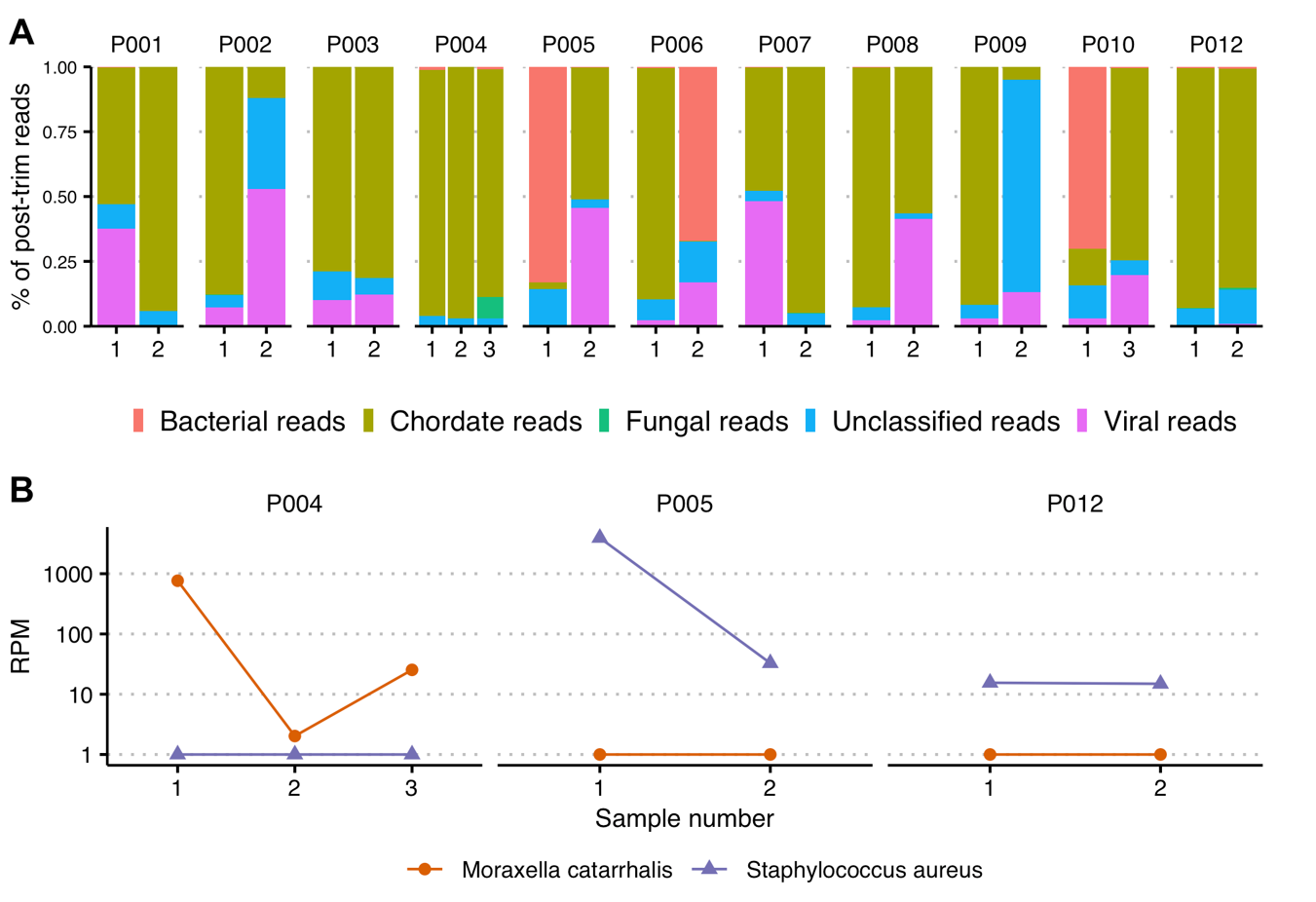
**

**Supplementary Figure S4.** **Post-trimmed reads used as input to metagenomic analysis.**

Samples with under 10,000 reads after trimming, and samples that underwent targeted sequencing (non-metagenomic) were excluded from downstream analysis.


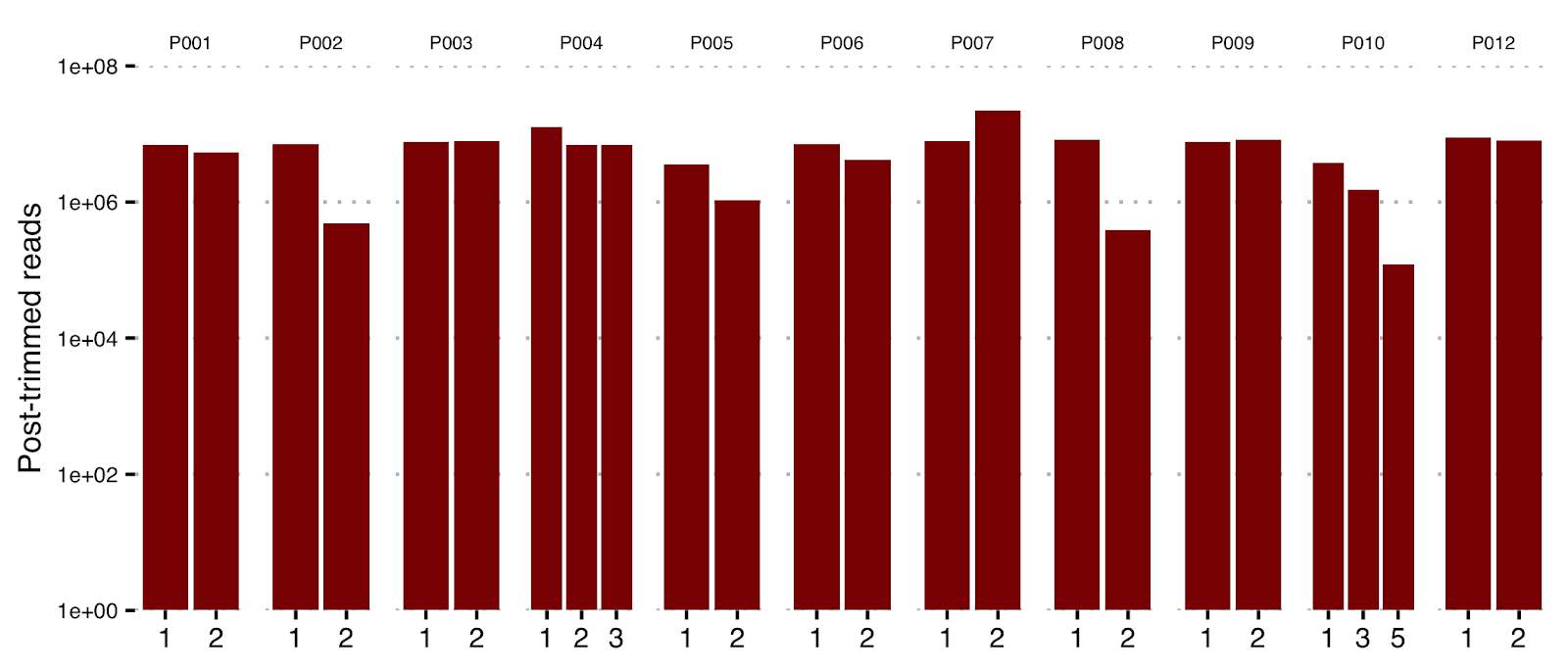


**Supplementary Table S4. Clinically relevant taxa**.

List of species and their respective NCBI taxonomic IDs deemed clinically relevant. Of these, *Moraxella catarrhalis* and *Staphylococcus aureus* were present in our dataset at significant RPMs.

| **Name** | **Rank** | **taxID** |
| --- | --- | --- |
| Adenoviridae | F | 10508 |
| Human coronavirus HKU1 | S | 290028 |
| Rousettus bat coronavirus HKU9 | S | 694006 |
| Human coronavirus NL63 | S | 277944 |
| Human coronavirus 229E | S | 11137 |
| Severe acute respiratory syndrome-related coronavirus | S | 694009 |
| Enterovirus | G | 12059 |
| Rhinovirus C | S | 463676 |
| Propionibacterium phage PacnesP2 | S | 1983621 |
| Propionibacterium phage pa33 | S | 2079406 |
| Propionibacterium phage Aquarius | S | 2041558 |
| Propionibacterium phage Moyashi | S | 1654781 |
| Propionibacterium phage PHL055N00 | S | 1500802 |
| Propionibacterium virus Pacnes201215 | S | 1982270 |
| Propionibacterium phage Pacnes 2012-15 | - | 1498188 |
| Streptococcus virus MS1 | S | 1962672 |
| environmental samples | - | 2100420 |
| uncultured Caudovirales phage | S | 2100421 |
| unclassified Papillomaviridae | - | 333774 |
| Human papillomavirus types | - | 173087 |
| Human papillomavirus | S | 10566 |
| Betacoronavirus BtCoV/Rhi_hip/R8-09/SPA/2010 | S | 1346312 |
| Bat Hp-betacoronavirus Zhejiang2013 | S | 2501961 |
| Bat Hp-betacoronavirus/Zhejiang2013 | - | 1541205 |
| Colobus guereza | S | 33548 |
| Influenza A virus | S | 11320 |
| Bordetella pertussis | S | 520 |
| Staphylococcus aureus | S | 1280 |
| Staphylococcus aureus | S | 1280 |
| Haemophilus influenzae | S | 727 |
| Pseudomonas aeruginosa | S | 287 |
| Pseudomonas oleovorans/pseudoalcaligenes group | - | 1232139 |
| Streptococcus pneumoniae | S | 1313 |
| Streptococcus sp. A12 | S | 1759399 |
| Streptococcus pseudopneumoniae | S | 257758 |
| Streptococcus pseudopneumoniae IS7493 | - | 1054460 |
| Streptococcus sp. I-G2 | S | 1156431 |
| Moraxella catarrhalis | S | 480 |
